# Effects of YM155 on survivin levels and viability in neuroblastoma cells with acquired drug resistance

**DOI:** 10.1038/cddis.2016.257

**Published:** 2016-10-13

**Authors:** Yvonne Voges, Martin Michaelis, Florian Rothweiler, Torsten Schaller, Constanze Schneider, Katharina Politt, Marco Mernberger, Andrea Nist, Thorsten Stiewe, Mark N Wass, Franz Rödel, Jindrich Cinatl

**Affiliations:** 1 Institut für Medizinische Virologie, Klinikum der Goethe-Universität, Paul Ehrlich-Str. 40, Frankfurt am Main 60596, Germany; 2 Centre for Molecular Processing and School of Biosciences, University of Kent, Canterbury CT2 7NJ, UK; 3 Institute of Molecular Oncology, Philipps-University, Marburg 35037, Germany; 4 Genomics Core Facility, Philipps-University, Marburg 35037, Germany; 5 Klinik für Strahlentherapie und Onkologie, Klinikum der Goethe-Universität, Theodor-Stern-Kai 7, Frankfurt am Main 60590, Germany

## Abstract

Resistance formation after initial therapy response (acquired resistance) is common in high-risk neuroblastoma patients. YM155 is a drug candidate that was introduced as a survivin suppressant. This mechanism was later challenged, and DNA damage induction and Mcl-1 depletion were suggested instead. Here we investigated the efficacy and mechanism of action of YM155 in neuroblastoma cells with acquired drug resistance. The efficacy of YM155 was determined in neuroblastoma cell lines and their sublines with acquired resistance to clinically relevant drugs. Survivin levels, Mcl-1 levels, and DNA damage formation were determined in response to YM155. RNAi-mediated depletion of survivin, Mcl-1, and p53 was performed to investigate their roles during YM155 treatment. Clinical YM155 concentrations affected the viability of drug-resistant neuroblastoma cells through survivin depletion and p53 activation. MDM2 inhibitor-induced p53 activation further enhanced YM155 activity. Loss of p53 function generally affected anti-neuroblastoma approaches targeting survivin. Upregulation of ABCB1 (causes YM155 efflux) and downregulation of SLC35F2 (causes YM155 uptake) mediated YM155-specific resistance. YM155-adapted cells displayed increased ABCB1 levels, decreased SLC35F2 levels, and a p53 mutation. YM155-adapted neuroblastoma cells were also characterized by decreased sensitivity to RNAi-mediated survivin depletion, further confirming survivin as a critical YM155 target in neuroblastoma. In conclusion, YM155 targets survivin in neuroblastoma. Furthermore, survivin is a promising therapeutic target for p53 wild-type neuroblastomas after resistance acquisition (neuroblastomas are rarely p53-mutated), potentially in combination with p53 activators. In addition, we show that the adaptation of cancer cells to molecular-targeted anticancer drugs is an effective strategy to elucidate a drug's mechanism of action.

Survivin, a member of the inhibitor of apoptosis protein (IAP) family, comprises a nodal protein implicated in a multitude of cellular pathways, including apoptosis and mitosis regulation, and is frequently found highly expressed in cancer cells, making it a potential target for anticancer therapies.^1, 2^ Indeed, a variety of survivin antagonists including YM155 entered clinical evaluation. YM155 (sepantronium bromide) was introduced as a transcriptional suppressor of survivin expression that displayed activity against a broad range of cancer types in preclinical models.^1, 3^ However, further studies suggested that the YM155-induced inhibition of survivin expression may be a secondary effect downstream of YM155-induced DNA damage^1, 4, 5^ or associated with Myeloid Cell Leukemia 1 (Mcl-1) depletion.^6^

Here we investigated the mechanism of action of YM155 in a panel consisting of the neuroblastoma cell lines UKF-NB-3 and UKF-NB-6 and their sublines with acquired resistance to cisplatin (UKF-NB-3^r^CDDP^1000^), doxorubicin (UKF-NB-6^r^DOX^20^), or vincristine (UKF-NB-3^r^VCR^10^ and UKF-NB-6^r^VCR^10^). Neuroblastoma is the most frequent solid extracranial pediatric cancer entity. About half of the patients are diagnosed with high-risk disease associated with overall survival rates below 50%, despite myeloablative therapy and differentiation therapy using retinoids.^7, 8^ Although many neuroblastomas respond initially well to therapy, acquired drug resistance represents a major obstacle in clinical practice.^7, 8^ Survivin had been previously shown to be a potential drug target in neuroblastoma.^9, 10, 11, 12, 13^ However, survivin had not been investigated as a therapeutic target in the acquired resistance setting in neuroblastoma prior to this study. Our principal findings are that survivin is a promising drug target in p53 wild-type neuroblastoma cells with acquired drug resistance and that YM155 impairs neuroblastoma cell viability in clinically achievable concentrations via survivin depletion. The drug-resistant cell lines displayed decreased sensitivity to YM155, with upregulation of the ATP-binding cassette (ABC) transporter ATP Binding Cassette Subfamily B Member 1 (ABCB1, also known as P-glycoprotein or multidrug resistance gene 1, MDR1; causes cellular YM155 efflux) and downregulation of Solute Carrier Family 35 Member F2 (SLC35F2, mediates cellular YM155 uptake) as the major drug-specific resistance mechanisms and loss of p53 function as resistance mechanism that affects all approaches targeting survivin in neuroblastoma. In accordance with these findings, neuroblastoma cells adapted to YM155 displayed decreased levels of SLC35F2, increased levels of ABCB1, a p53 mutation, decreased levels of survivin, and decreased sensitivity to RNAi-mediated survivin depletion.

## Results

### Effects of YM155 on neuroblastoma cell viability

**Figure 1:**
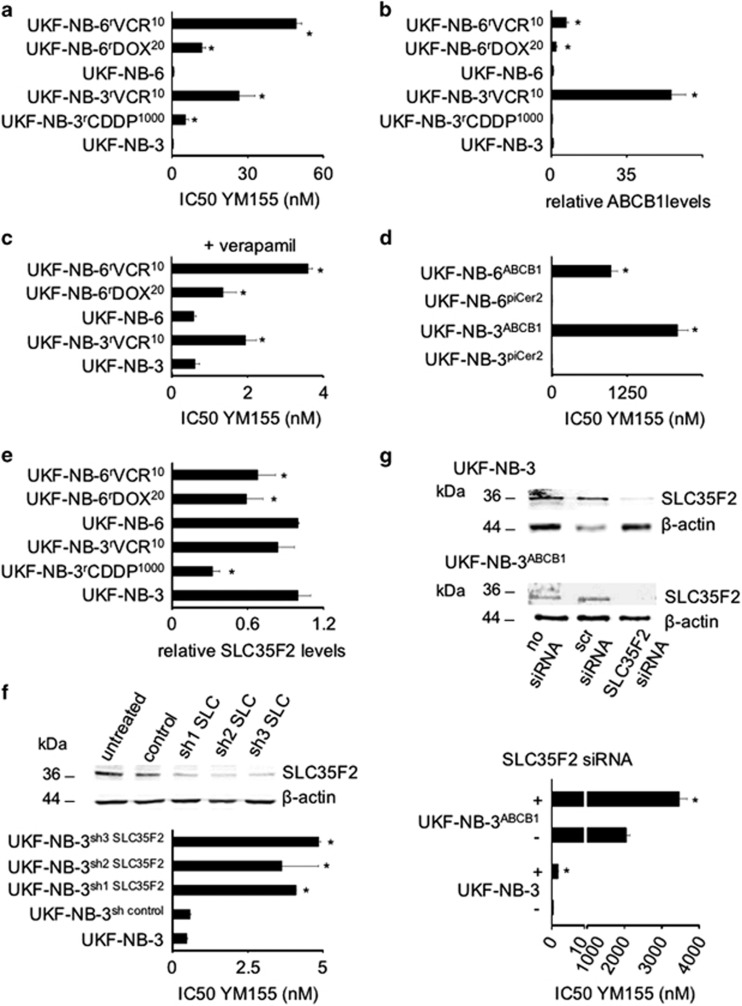
Effects of YM155 on neuroblastoma cell viability and the role of ABCB1 and SLC35F2 expression. (**a**) YM155 concentrations that reduce the viability of the investigated neuroblastoma cell lines by 50% (IC50) as determined by MTT assay after a 120 h incubation period (numerical values are presented in Supplementary Table 1). *P<0.05 relative to the respective parental cell line; (**b**) Cellular ABCB1 levels as indicated by quantitative, fluorescence-based western blot analysis; values are presented as fold change relative to the respective parental cell line. Representative western blots are provided in Supplementary Figure 2. *P<0.05 relative to the respective parental cell line; (**c**) YM155 IC50s as determined by MTT assay after a 120 h incubation period in the presence of the ABCB1 inhibitor verapamil (5 *μ*M) in the parental cell lines UKF-NB-3 and UKF-NB-6 and their ABCB1-expressing sublines UKF-NB-3^r^VCR^10^, UKF-NB-6^r^DOX^20^, and UKF-NB-6^r^VCR^10^ (numerical values are presented in Supplementary Table 1). *P<0.05 relative to the respective parental cell line; (**d**) YM155 IC50s as determined by MTT assay after a 120 h incubation in UKF-NB-3 or UKF-NB-6 cells transduced with a lentiviral vector encoding for ABCB1 or an empty control vector (piCER2). *P<0.05 relative to the respective control vector-transduced cell line; (**e**) Cellular SLC35F2 levels as indicated by quantitative, fluorescence-based western blot analysis; values are presented as fold change relative to the respective parental cell line. Representative western blots are provided in Supplementary Figure 2. *P<0.05 relative to the respective parental cell line; (**f**) Western blot indicating SLC35F2 depletion in UKF-NB-3 cells by three lentiviral vectors encoding for shRNA directed against three different sequences of SLC35F2 (sh1 SLC, sh2 SLC, sh3 SLC) relative to untreated and empty control vector-treated (control) UKF-NB-3 cells and bar chart indicating YM155 concentrations that reduce the viability of these cell lines by 50% (IC50) as indicated by MTT assay after 120 h of incubation. Numerical IC50 values are presented in Supplementary Table 2. *P<0.05 relative to non-treated cells; (**g**) Western blots indicating siRNA-mediated SLC35F2 depletion (scr siRNA, non-targeting ‘scrambled' siRNA) and bar chart displaying YM155 IC50 values (as indicated by MTT assay after 120 h of incubation) in UKF-NB-3 and ABCB1-transduced UKF-NB-3 (UKF-NB-3^ABCB1^) cells with or without transfection by siRNA directed against SLC35F2. Numerical values and controls transfected with non-targeting siRNA are presented in Supplementary Table 3. *P<0.05 relative to the respective non-transfected cells

Treatment of the neuroblastoma cell lines UKF-NB-3 and UKF-NB-6 with YM155 resulted in IC50 values of 0.49 and 0.65 nM, respectively (Figure 1a and Supplementary Table 1). The UKF-NB-3 sublines with acquired resistance to cisplatin (UKF-NB-3^r^CDDP^1000^) or vincristine (UKF-NB-3^r^VCR^10^), as well as the UKF-NB-6 sublines resistant to doxorubicin (UKF-NB-6^r^DOX^20^) or vincristine (UKF-NB-6^r^VCR^10^), displayed substantially reduced YM155 sensitivity compared to the parental cell lines, resulting in IC50 values ranging from 5.32 nM (UKF-NB-3^r^CDDP^1000^) to 49.3 nM (UKF-NB-6^r^VCR^10^) (Figure 1a and Supplementary Table 1). There was no correlation between the YM155 IC50 and the survivin expression levels (Supplementary Figure 1).

### ABCB1 expression decreases YM155 efficacy in neuroblastoma cells

ABCB1 expression was previously shown to interfere with YM155 efficacy.^10, 14^ Among the investigated cell lines, UKF-NB-3^r^VCR^10^, UKF-NB-6^r^DOX^20^, and UKF-NB-6^r^VCR^10^ display detectable ABCB1 activity (Figure 1b and Supplementary Figure 2). In accordance, the ABCB1 inhibitor verapamil reduced the YM155 IC50s in these cell lines (Figure 1c and Supplementary Table 1). Furthermore, UKF-NB-3 and UKF-NB-6 cells transduced with a lentiviral vector encoding for ABCB1 demonstrated strongly increased resistance to YM155 (Figure 1d and Supplementary Figure 2). However, treatment of the ABCB1-expressing cell lines UKF-NB-3^r^VCR^10^, UKF-NB-6^r^DOX^20^, and UKF-NB-6rVCR^10^ with the ABCB1 inhibitor verapamil did not reduce the YM155 IC50s to the level of parental UKF-NB-3 or UKF-NB-6 cells (Figure 1c and Supplementary Table 1). This indicates that other mechanisms in addition to ABCB1 expression interfere with YM155 efficacy in the resistant cell lines.

### Reduced SLC35F2 levels contribute to decreased YM155 sensitivity in drug-resistant neuroblastoma cells

The solute carrier SLC35F2 was shown to mediate cellular uptake of YM155.^15^ Reduced SLC35F2 levels were detected in UKF-NB-3^r^CDDP^1000^, UKF-NB-6^r^DOX^20^, and UKF-NB-6^r^VCR^10^ cells (Figure 1e and Supplementary Figure 2). Hence, decreased SLC35F2 levels appear to contribute to YM155 resistance in these cell lines. In accordance, RNAi-mediated depletion of SLC35F2 reduced the YM155 sensitivity of UKF-NB-3 cells (Figure 1f and Supplementary Table 2) and further increased the YM155 resistance of ABCB1-transduced UKF-NB-3 and UKF-NB-6 cells (Figure 1g and Supplementary Table 3).

### p53 status influences neuroblastoma cell sensitivity to YM155

**Figure 2:**
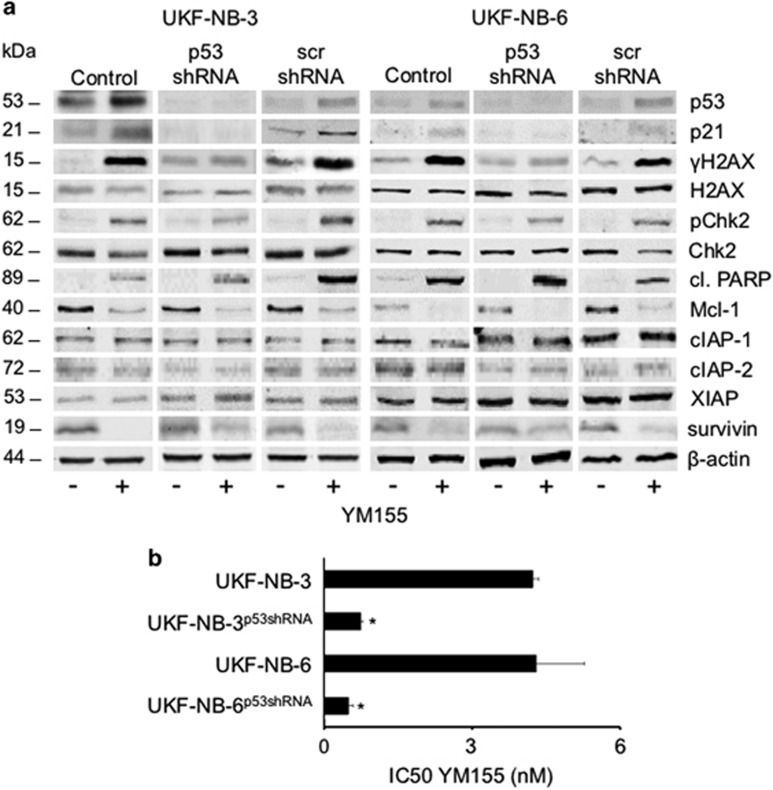
Relevance of functional p53 for the YM155-mediated anti-neuroblastoma effects. (**a**) Protein levels of p53, p21, *γ*H2AX, H2AX, phosphorylated Chk2 (pChk2), Chk2, cleaved PARP (cl. PARP), Mcl-1, cIAP-1, cIAP-2, XIAP, and survivin after YM155 (25 nM) treatment for 24 h of non-transduced UKF-NB-3/UKF-NB-6 cells (Control), UKF-NB-3/UKF-NB-6 cells transduced with a lentiviral vector encoding for shRNA targeting p53 (p53 shRNA), or UKF-NB-3/UKF-NB-6 cells transduced by a control vector encoding for non-targeting (‘scrambled') shRNA (scr shRNA); *β*-actin served as loading control. (**b**) YM155 concentrations that reduce neuroblastoma cell viability by 50% (IC50) in UKF-NB-3 and UKF-NB-6 cells in the absence or presence of functional p53; p53 was depleted using a lentiviral vector encoding for shRNA targeting p53 (UKF-NB-3^p53shRNA^ and UKF-NB-6^p53shRNA^). Numerical values and control cell lines transduced with non-targeting shRNA are presented in Supplementary Table 4. *P<0.05 relative to the respective non-transduced cell

In contrast to the other ABCB1-expressing neuroblastoma cell lines UKF-NB-6^r^DOX^20^ and UKF-NB-6^r^VCR^10^, the vincristine-resistant UKF-NB-3 subline UKF-NB-3^r^VCR^10^ did not display decreased levels of SLC35F2 (Figure 1e), although it was not resensitized to YM155 to the level of the respective parental cell line UKF-NB-3 by the ABCB1 inhibitor verapamil (Figure 1b and Supplementary Table 1). An important difference between UKF-NB-3^r^VCR^10^ and the other investigated neuroblastoma cell lines is that UKF-NB-3^r^VCR^10^ cells harbor a C135F p53 mutation.^16, 17, 18^ Indeed, YM155 induced a p53 response in UKF-NB-3 and UKF-NB-6 cells as indicated by increased cellular levels of p53 and p21, and apoptosis as indicated by poly(ADP-ribose) polymerase (PARP) cleavage (Figure 2a). Moreover, p53-depleted UKF-NB-3 and UKF-NB-6 cells displayed no activation of p53 signaling and substantially decreased sensitivity in response to YM155 (Figures 2a and b and Supplementary Table 4). Similar results were obtained for the nutlin-3-resistant UKF-NB-3 subline UKF-NB-3^r^Nutlin^10μM^ (Supplementary Table 4) that harbors a G245C loss-of-function p53 mutation.^18^

**Figure 3:**
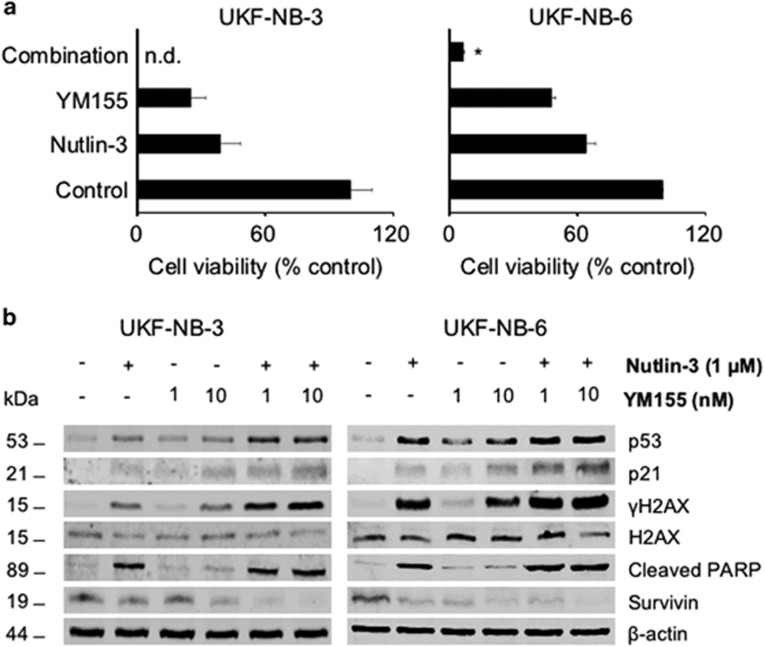
Effects of p53 activation by the MDM2 inhibitor nutlin-3 on the YM155-induced effects on neuroblastoma cell viability. (**a**) Effects of nutlin-3 (1 *μ*M), YM155 (0.625 nM), or their combination on neuroblastoma cell viability as indicated by MTT assay after 120 h incubation. *P<0.05 relative to either single treatment; n.d.=no detectable cell viability; (**b**) Representative western blots indicating the effects of combined YM155 and nutlin-3 treatment on p53 signaling (p53 and p21 levels), DNA damage (*γ*H2AX levels), and survivin levels in UKF-NB-3 and UKF-NB-6 cells after 24 h of incubation. The respective quantitative data are provided in Supplementary Figure 3

In order to determine whether additional activation of p53 may further increase YM155 activity, we combined YM155 with nutlin-3 that activates p53 through Mouse Double Minute 2 (MDM2) inhibition.^19^ Indeed, nutlin-3 increased the anticancer effects of YM155 (Figure 3a) and p53 signaling (Figure 3b and Supplementary Figure 3), suggesting that p53 activation plays a pivotal role within the anticancer effects of YM155.

### YM155 interferes with neuroblastoma cell viability through survivin depletion

The anticancer mechanism of action of YM155 is under dispute. The compound was introduced as a suppressor of survivin expression. However, this mechanism was challenged and some studies suggested that the YM155-induced inhibition of survivin expression may be a secondary effect downstream of YM155-induced DNA damage.^1, 3, 4^

**Figure 4:**
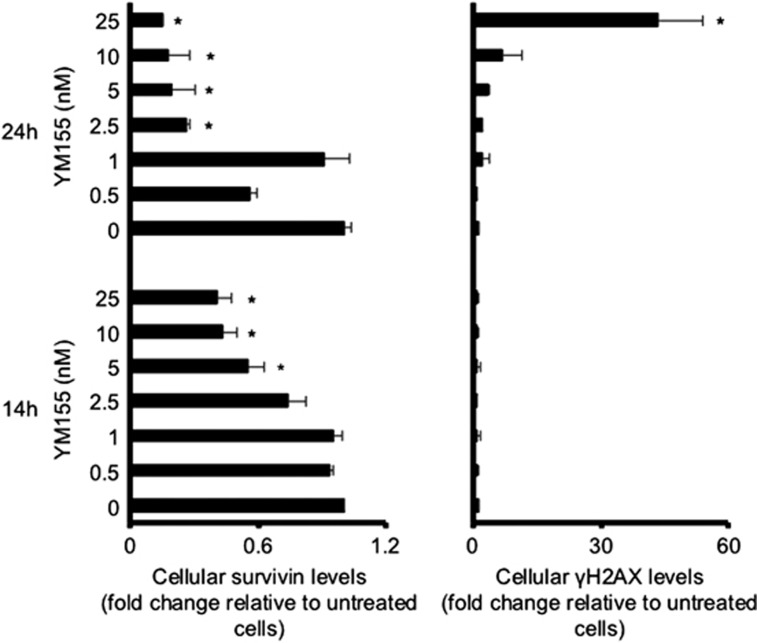
Kinetics of survivin depletion and DNA damage induction by YM155 in neuroblastoma cells. Levels of survivin and *γ*H2AX (indicating DNA damage) in UKF-NB-3 cells in response to YM155 treatment after 14 or 24 h as indicated by quantitative, fluorescence-based western blot analysis; values are presented as fold change relative to the respective parental cell line. Detailed kinetics and representative western blots are provided in Supplementary Figure 4. *P<0.05 relative to untreated control

**Figure 5:**
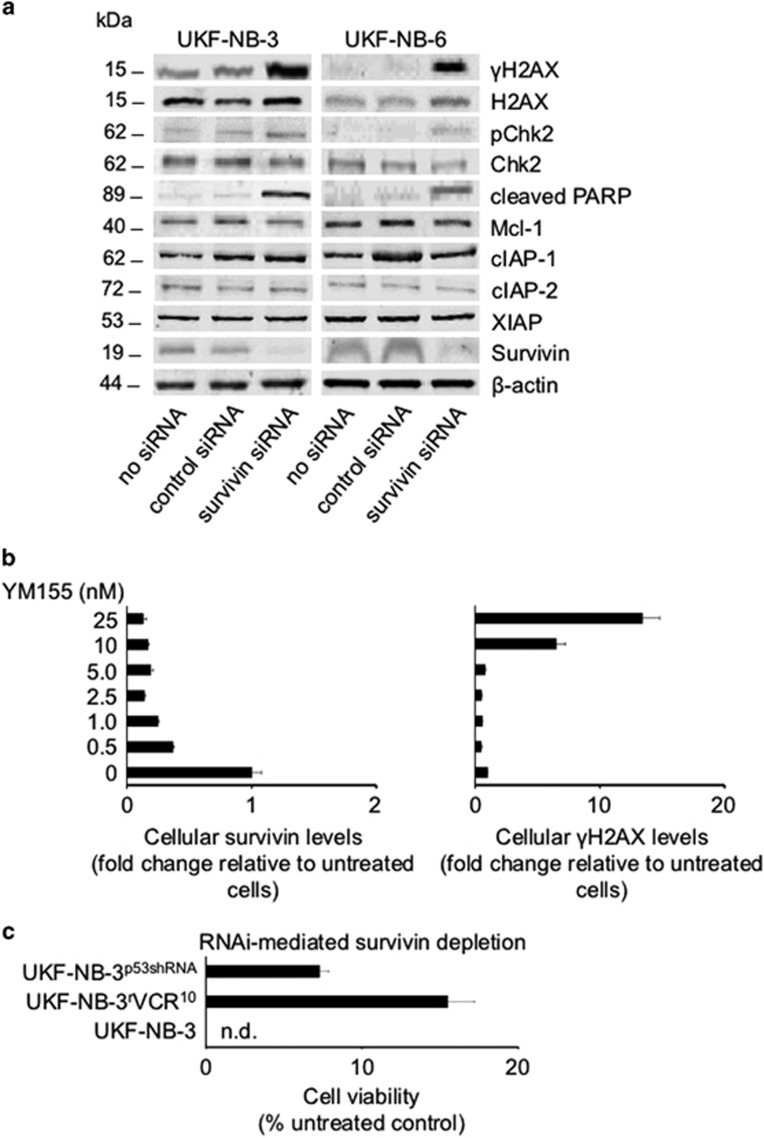
Effects of siRNA-mediated survivin depletion on neuroblastoma cell viability. (**a**) Western blots indicating levels of *γ*H2AX, H2AX, phosphorylated Chk2 (pChk2), Chk2, cleaved PARP, Mcl-1, cIAP-1, cIAP-2, XIAP, or survivin in neuroblastoma cells, neuroblastoma cells transfected with non-targeting control siRNA, and neuroblastoma cells transfected with siRNA targeting survivin (BIRC5) 24 h post transfection. *β*-actin served as loading control. (**b**) Levels of survivin and *γ*H2AX (indicating DNA damage) in UKF-NB-3 cells transduced with a lentiviral vector encoding for shRNA targeting p53 (UKF-NB-3^p53shRNA^) in response to YM155 treatment for 48 h as indicated by quantitative, fluorescence-based western blot analysis; values are presented as fold change relative to the respective parental cell line. Representative western blots are provided in Supplementary Figure 4. (**c**) Viability of UKF-NB-3 cells, UKF-NB-3^r^VCR^10^ cells, and UKF-NB-3^p53shRNA^ cells 48 h after transfection with siRNA targeting survivin (BIRC5); Western blots indicating depletion efficiency in UKF-NB-3^r^VCR^10^ cells and UKF-NB-3^p53shRNA^ cells are presented in Supplementary Figure 11. Numerical values including controls transfected with non-targeting siRNA are shown in Supplementary Table 5

**Figure 6:**
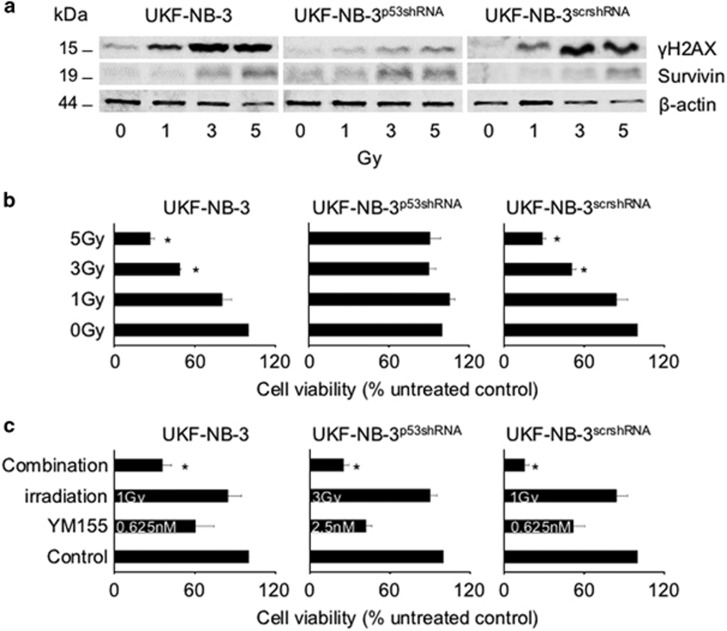
Role of survivin in response to irradiation-induced DNA damage in neuroblastoma cells. (**a**) Representative western blots indicating cellular levels of *γ*H2AX and survivin 24 h after irradiation of UKF-NB-3 cells, UKF-NB-3 cells transduced with a lentiviral vector encoding shRNA directed against p53 (UKF-NB-3^p53shRNA^), or UKF-NB-3 cells transduced with a control vector encoding non-targeting (‘scrambled') shRNA (UKF-NB-3^scrshRNA^). *β*-actin served as a loading control. Quantitative, fluorescence-based western blot analyses are presented in Supplementary Figure 6. (**b**) Viability of UKF-NB-3, UKF-NB-3^p53shRNA^, or UKF-NB-3^scrshRNA^ cells 24 h post irradiation as indicated by MTT assay; *P 0.05 relative to untreated control cells; (**c**) Combined effects of irradiation and YM155 on UKF-NB-3 (1 Gy, YM155 0.625 nM), UKF-NB-3^p53shRNA^ (3 Gy, YM155 2.5 nM), or UKF-NB-3^scrshRNA^ (1 Gy, YM155 0.625 nM) cell viability 24 h post irradiation as indicated by MTT assay; *P 0.05 relative to either single treatment

In order to determine the mechanism of action of YM155 in our model system, we determined the kinetics of cellular survivin levels and DNA damage in response to YM155 treatment over time. Although survivin depletion became visible after 14 h of treatment at an YM155 concentration of 5 nM, a substantial increase of the phosphohistone *γ*-histone H2A (*γ*H2AX) levels indicating DNA double-strand breaks became only detectable after 24 h at YM155 concentrations of 10 nM or higher (Figure 4 and Supplementary Figure 4). In accordance, a DNA damage kit that determines apurinic/apyrimidinic sites as indicators of DNA lesions by using an Aldehyde Reactive Probe (PromoKine, Heidelberg, Germany) did only detect DNA damage in UKF-NB-3 cells after 24 h of YM155 (10 nM) treatment but not after 14 h of YM155 (10 nM) treatment (Supplementary Figure 5). This shows that YM155-induced suppression of survivin expression precedes DNA damage formation. RNAi-mediated survivin depletion resulted in an increase of *γ*H2AX levels and Checkpoint Kinase 2 (Chk2) phosphorylation in UKF-NB-3 and UKF-NB-6 cells (Figure 5a), demonstrating that survivin depletion is sufficient to induce DNA damage in our system. The notion that YM155-induced survivin depletion is not a consequence of DNA damage induction in neuroblastoma cells was further supported by data showing that irradiation-induced DNA damage did not decrease the survivin levels in UKF-NB-3 cells but rather resulted in enhanced survivin levels, both in the presence and in the absence of p53 (Figure 6a and Supplementary Figure 6). p53-depleted UKF-NB-3 cells were substantially more resistant to irradiation than UKF-NB-3 cells (Figure 6b), but, notably, YM155 increased the anti-neuroblastoma effects of irradiation in UKF-NB-3 cells and p53-depleted UKF-NB-3 cells (Figure 6c).

Neuroblastoma cells without functional p53 had been shown to be less sensitive to YM155 than neuroblastoma cells with functional p53 (Figures 2 and 3). In concordance, YM155 treatment resulted in delayed survivin depletion and DNA double-strand break formation in p53-depleted UKF-NB-3 cells compared to wild-type p53-expressing UKF-NB-3 cells (Supplementary Figures 4 and 7). Reduced survivin levels became detectable in p53-depleted UKF-NB-3 cells only after 18–24 h of YM155 treatment. DNA damage as indicated by *γ*H2AX levels was (in contrast to YM155-treated UKF-NB-3 cells) not detected after 24 h treatment of p53-depleted UKF-NB-3 cells with YM155 concentrations of up to 25 nM (Supplementary Figures 4 and 7). YM155-induced survivin depletion became visible in p53-depleted UKF-NB-3 cells only after 48 h of YM155 treatment (Figure 5b and Supplementary Figure 8). DNA damage induction was only detected at YM155 concentrations of 10 and 25 nM after 48 h of treatment (Figure 5b and Supplementary Figure 8). In accordance, p53-depleted UKF-NB-3 cells and p53-mutant UKF-NB-3^r^VCR^10^ were less sensitive to siRNA-mediated survivin depletion than UKF-NB-3 cells (Figure 5c and Supplementary Table 5).

Mcl-1 was suggested to be an additional target of YM155.^6^ Compared to YM155-mediated survivin depletion, we detected reduced Mcl-1 levels only in response to substantially higher YM155 concentrations and at later time points (Supplementary Figure 9). Moreover, Mcl-1 depletion did (in contrast to survivin depletion) not affect UKF-NB-3 cell viability (Supplementary Figure 10), suggesting that YM155-mediated survivin depletion is critical for the compound's effects on neuroblastoma cell viability. The Mcl-1 levels are not regulated by survivin since survivin depletion did not decrease the Mcl-1 levels in UKF-NB-3 and UKF-NB-6 cells (Figure 5a).

Taken together, these findings demonstrate that survivin is a critical target in neuroblastoma cells, that the anti-neuroblastoma effects of YM155 involve survivin depletion (prior to DNA damage induction), and that p53 is involved in mediating anti-neuroblastoma effects as a consequence of RNAi- or YM155-induced reduction of cellular survivin levels.

### Neuroblastoma cells adapted to YM155 display reduced survivin and SLC35F2 levels, increased ABCB1 levels, a p53 mutation, and decreased sensitivity to survivin depletion

**Figure 7:**
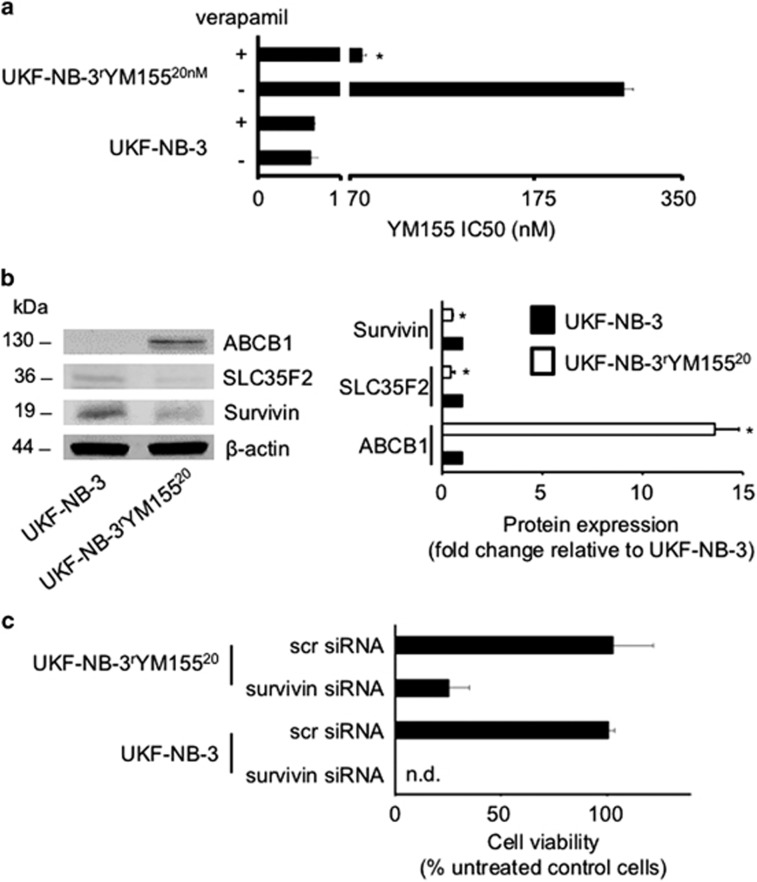
Characterization of UKF-NB-3 cells that were adapted to growth in the presence of YM155 20 nM (UKF-NB-3^r^YM155^20nM^). (**a**) YM155 concentrations that reduce UKF-NB-3 cell viability by 50% (IC50) in the presence or absence of the ABCB1 inhibitor verapamil (5 *μ*M). Numerical values are presented in Supplementary Table 6. *P<0.05 relative to the YM155 IC50 in the absence of verapamil; (**b**) Representative western blots and fluorescence-based quantitative western blot analysis data indicating cellular protein levels of ABCB1, SLC35F2, and survivin in UKF-NB-3 and UKF-NB-3^r^YM155^20nM^ cells. *P<0.05 relative to UKF-NB-3 cells; (**c**) Cell viability in UKF-NB-3 and UKF-NB-3^r^YM155^20nM^ cells 48 h after transfection with siRNA directed against survivin or non-targeting ‘scrambled' (scr) siRNA relative to untreated control cells. Numerical data are presented in Supplementary Table 5. n.d.=non-detectable

Finally, we established a YM155-resistant UKF-NB-3 cell line (UKF-NB-3^r^YM155^20nM^). UKF-NB-3^r^YM155^20nM^ cells displayed a 497-fold increased resistance to YM155 (IC50: 303±8 nM) compared to parental UKF-NB-3 cells (IC50: 0.61±0.08 nM) (Figure 7a and Supplementary Table 6). Moreover, UKF-NB-3^r^YM155^20nM^ cells expressed increased levels of ABCB1, decreased levels of SLC35F2 (Figure 7b), and harbored a R248W p53 mutation. This suggests that multiple resistance mechanisms contribute to the acquired YM155 resistance phenotype observed in these cells. In accordance, the ABCB1 inhibitor verapamil (5 *μ*M) sensitized UKF-NB-3^r^YM155^20nM^ cells to YM155 (IC50: 90.5±3.1 nM) but not to the level of parental UKF-NB-3 cells (Figure 7a and Supplementary Table 6).

UKF-NB-3^r^YM155^20nM^ cells also displayed reduced survivin levels (Figure 7b) and reduced sensitivity to survivin depletion relative to UKF-NB-3 cells (Figure 7c and Supplementary Table 5). This further confirmed that survivin is a primary target of YM155 in neuroblastoma.

## Discussion

Here we investigated the efficacy of the survivin suppressant YM155 in a panel of neuroblastoma cell lines consisting of the V-Myc Avian Myelocytomatosis Viral Oncogene Neuroblastoma Derived Homolog (MYCN)-amplified neuroblastoma cell lines UKF-NB-3 und UKF-NB-6 and their sublines with acquired resistance to cisplatin (UKF-NB-3^r^CDDP^1000^), doxorubicin (UKF-NB-6^r^DOX^20^), and vincristine (UKF-NB-3^r^VCR^10^ and UKF-NB-6^r^VCR^10^). The YM155 IC50 values ranged from 0.49 nM (UKF-NB-3) to 49.3 nM (UKF-NB-6^r^VCR^10^). This is in the range of therapeutically achievable plasma levels that were documented to reach about 50 nM.^20, 21, 22^ Notably, animal models indicated that the YM155 concentrations in tumor tissues are likely to be higher than those found in the plasma.^3, 23^ Previously, YM155 IC50 values were reported to range from 3.2 to 11 nM in melanoma cells^24^ and from 2 to 50 nM in multiple myeloma cells.^25^ In a screen of 124 cell lines from 17 cancer entities, the YM155 IC50s ranged from 0.35 to >1000 nM.^26^ In a panel of 23 neuroblastoma cell lines, the YM155 IC50s ranged from 0.5 to >10 000 nM.^10^ This shows that the sensitivity to YM155-mediated survivin depletion may substantially vary even in cells from the same cancer type. Hence, it will be critical to understand which cancer cells are particularly sensitive to YM155 and survivin depletion in order to design tailored therapies.

The parental cell lines UKF-NB-3 and UKF-NB-6 displayed a similar response to YM155 treatment. The YM155 IC50s were comparable (UKF-NB-3: 0.49±0.10 nM; UKF-NB-6: 0.65±0.09 nM), and YM155 induced survivin depletion and p53 induction in both cell lines. All investigated drug-resistant sublines of UKF-NB-3 and UKF-NB-6 showed substantially reduced YM155 sensitivity relative to the parental cell lines. Three mechanisms may explain the decreased YM155 sensitivity in the drug-resistant cancer cell lines (at least to a large degree): upregulation of ABCB1, downregulation of SLC35F2, and loss of p53 function. Upregulation of the ABC transporter ABCB1 had already been shown to affect the efficacy of YM155.^10, 14^ In accordance, the ABCB1 inhibitor verapamil strongly decreased the YM155 IC50 values in the ABCB1-expressing cell lines UKF-NB-3^r^VCR^10^ (from 26.59 to 1.95 nM), UKF-NB-6^r^DOX^20^ (from 11.80 to 1.35 nM), and UKF-NB-6^r^VCR^10^ (from 49.30 to 3.60 nM), but not to the level of the parental cell lines that displayed IC50 values of 0.49 nM (UKF-NB-3) and 0.65 nM (UKF-NB-6). Hence, additional resistance mechanisms appear to contribute to the decreased YM155 sensitivity that was observed in the drug-resistant neuroblastoma sublines in comparison to the respective parental chemosensitive cells.

YM155 is transported by SLC35F2 into cells and lack of SLC35F2 has been associated with decreased cellular YM155 sensitivity.^15^ The drug-resistant neuroblastoma cell lines UKF-NB-3^r^CDDP^1000^, UKF-NB-6^r^DOX^20^, and UKF-NB-6^r^VCR^20^ were characterized by reduced SLC35F2 expression relative to the respective parental cells. Hence, these decreased SLC35F2 levels are likely to contribute to the increased YM155 resistance observed in these cell lines when compared to the respective parental cell lines. This notion is further supported by our finding that SLC35F2 depletion increases the resistance of ABCB1-expressing neuroblastoma cells. Since SLC35F2 and ABCB1 affect intracellular YM155 concentrations, these represent drug-specific resistance mechanisms that may not be shared by other therapeutic strategies that target survivin.

UKF-NB-3^r^VCR^10^ was the only drug-resistant cell line that did not display decreased SLC35F2 levels. However, ABCB1 inhibition did not resensitize UKF-NB-3^r^VCR^10^ cells to YM155 to the level of the parental UKF-NB-3 cell line. In contrast to the other investigated cell lines, UKF-NB-3^r^VCR^10^ harbors a C135F loss-of-function p53 mutation.^16, 17, 18^ Hence, loss of p53 function may impair the YM155 sensitivity of neuroblastoma cells. Indeed, p53 depletion resulted in decreased YM155 sensitivity of UKF-NB-3 and UKF-NB-6 cells. Moreover, the nutlin-3-resistant UKF-NB-3 subline UKF-NB-3^r^Nutlin^10μM^ that harbors a G245C loss-of-function p53 mutation^18^ displayed reduced sensitivity to YM155 relative to UKF-NB-3. Other reports had suggested that YM155 exerts its anticancer effects independently of the cellular p53 status.^26, 27, 28^ However, the relevance of the p53 status for cancer cell sensitivity to YM155 and survivin depletion may depend on the cellular context.

The anticancer mechanism of action of YM155 has been under dispute and may depend on the cellular background. YM155 was introduced as a compound that suppresses the expression of survivin.^26^ However, other studies suggested that YM155 may primarily induce DNA damage and that the reduction of survivin levels may rather be a consequence of DNA damage.^1, 3, 4^ In our study, however, survivin downregulation preceded the occurrence of DNA damage in response to YM155 treatment, suggesting that survivin depletion happens prior to DNA damage formation. In accordance, RNAi-mediated survivin depletion also resulted in the induction of DNA damage, demonstrating that DNA damage formation can be a consequence of the reduction of cellular survivin levels. Notably, we and others had previously shown that survivin depletion resulted in increased yH2AX foci detection following irradiation^29, 30^ that is functionally related to a direct physical interrelationship of survivin with components of DNA damage repair pathways.^31, 32^

Mcl-1 was suggested as an alternative target of YM155.^6^ However, YM155 reduced cellular Mcl-1 levels at substantially higher concentrations than survivin, and Mcl-1 depletion did not affect neuroblastoma cell viability. The finding that Mcl-1 depletion does not impair neuroblastoma cell viability is in accordance with previous results demonstrating that Mcl-1 depletion sensitizes neuroblastoma cells to paclitaxel, but does not exert significant effects on neuroblastoma cell viability on its own.^33^ Hence, the anti-neuroblastoma effects of YM155 do not appear to be caused by Mcl-1 depletion. Finally, survivin depletion-mediated X-linked inhibitor of apoptosis (XIAP) degradation had been shown to contribute to the effects of YM155 against breast cancer cells.^34^ However, survivin depletion did not decrease XIAP levels in our models (Figures 2 and 5).

Neuroblastoma cells without functional p53 displayed not only reduced sensitivity to YM155 but also to RNAi-mediated survivin depletion. This suggests that loss of p53 function is a target-specific resistance mechanism that is likely to generally affect the efficacy of therapeutic approaches targeting survivin. A role of p53 in the cellular response to survivin depletion may not come too much as a surprise, given the close interplay of survivin and p53.^1, 35^ For example, survivin depletion caused p53 activation in neuroblastoma cells not only in our study but also in a previous study.^9^ In ALL cells, survivin depletion-induced toxicity could be rescued by p53.^36^

Next, UKF-NB-3 cells were adapted to growth in the presence of YM155 (20 nM, UKF-NB-3^r^YM155^20nM^) and the resistance-associated changes were investigated. We detected a R248W p53 mutation as target-specific resistance mechanism, and decreased SLC35F2 and increased ABCB1 as drug-specific resistance mechanisms. UKF-NB-3^r^YM155^20nM^ cells also displayed decreased survivin levels and sensitivity to RNAi-mediated survivin depletion relative to UKF-NB-3, further confirming that YM155 primarily exerts its anti-neuroblastoma effects through survivin depletion.

In conclusion, our data show that YM155 affects the viability of neuroblastoma cells, including neuroblastoma cells with acquired resistance to clinically relevant drugs through survivin depletion and that survivin is a promising drug target in neuroblastoma cells with acquired drug resistance. Cells adaptated to YM155 displayed decreased survivin levels and decreased sensitivity to RNAi-mediated survivin depletion, confirming that YM155 targets survivin. Thus, cancer cell adaptation to anticancer drugs represents a novel strategy to elucidate the drug's mechanism of action. Loss of p53 function was determined as target-specific resistance mechanism that generally affects therapeutic strategies directed against survivin. Hence, survivin is particularly promising as drug target for the treatment of p53 wild-type neuroblastomas, including neuroblastomas with acquired resistance to clinically relevant anticancer drugs, potentially in combination with p53 activators such as MDM2 inhibitors that further enhance YM155 activity. Notably, p53 wild-type neuroblastomas make up the vast majority of neuroblastoma cases, as neuroblastomas only rarely harbor p53 mutations.^37, 38^

## Materials and Methods

### Drugs

YM155 (sepantronium bromide) was purchased from Selleck Chemicals via BIOZOL Diagnostica GmbH (Eching, Germany), vincristine and cisplatin from TEVA GmbH (Radebeul, Germany), doxorubicin from Cell Pharm (Bad Vilbel, Germany), and verapamil from Sigma-Aldrich (Munich, Germany).

### Cells

The MYCN-amplified neuroblastoma cell lines UKF-NB-3 and UKF-NB-6 were established from stage 4 neuroblastoma patients.^16, 39^ UKF-NB-3 and UKF-NB-6 sublines with acquired resistance to cisplatin (UKF-NB-3^r^CDDP^1000^), doxorubicin (UKF-NB-6^r^DOX^20^), vincristine (UKF-NB-3^r^VCR^10^ and UKF-NB-6^r^VCR^10^), or nutlin-3 (UKF-NB-3^r^Nutlin^10μM^) were established by continuous exposure to increasing drug concentrations as described previously^16, 18, 39^ and derived from the Resistant Cancer Cell Line collection (www.kent.ac.uk/stms/cmp/RCCL/RCCLabout.html).

All cells were propagated in Iscove's modified Dulbecco's medium supplemented with 10% fetal calf serum, 100 IU/ml penicillin, and 100 mg/ml streptomycin at 37°C. Cells were routinely tested for mycoplasma contamination and authenticated by short tandem repeat profiling.

p53-depleted neuroblastoma cells or neuroblastoma cells showing high expression of ABCB1 were established as described previously^39, 40, 41^, using the Lentiviral Gene Ontology (LeGO) vector technology (www.lentigo-vectors.de).^42^

SLC35F2-depleted cells were established using the same technology. The shRNA sequences (5′ to 3′) were sh1 SLC35F2, CTCTTTCTGTTTGGCTATA; sh2 SLC35F2, GAGGAATACATCGTGAAGA; sh3 SLC35F2, CAGGGAGTGATGTATTGAT.

### Viability assay

Cell viability was determined by the 3-(4,5-dimethylthiazol-2-yl)-2,5-diphenyltetrazolium bromide (MTT) dye reduction assay after 120 h incubation, modified as described previously.^18, 39, 41^

### RNA interference experiments

Transient depletion of SLC35F2 was achieved using synthetic siRNA oligonucleotides (ON-TARGETplus SMARTpool) from Dharmacon (Lafayette, CO, USA). Non-targeting siRNA (ON-TARGETplus SMARTpool) was used as negative control. Cells were transfected by electroporation using the NEON Transfection System (Invitrogen, Darmstadt, Germany) according to the manufacturer protocol. Cells were grown to 60–80% confluence, trypsinized, and 1.2 × 10^6^ cells were resuspended in 200 *μ*l resuspension buffer R including 2.5 *μ*M siRNA. The electroporation was performed using two 20 ms pulses of 1400 V. Subsequently, the cells were transferred into cell culture plates or flasks, containing prewarmed cell culture medium.

### Western blot

Cells were lysed using Triton-X-100 sample buffer, and proteins were separated by SDS-PAGE. Detection was done by using specific antibodies against *β*-actin (Biovision through BioCat GmbH, Heidelberg, Germany), SLC35F2 (Santa Cruz Biotechnology, Dallas, TX, USA), ABCB1, Chk2, phosphorylated Chk2 (Thr68), Baculoviral IAP repeat containing 3 (cIAP-2), histone H2AX, phosphorylated histone H2AX (Ser139) (*γ*H2AX), myeloid cell leukemia sequence-1 (Mcl-1), p21, cleaved PARP, XIAP (all from Cell Signaling via New England Biolabs, Frankfurt, Germany), p53 (Enzo Life Sciences, Lörrach, Germany), survivin and Baculoviral IAP repeat containing 2 (cIAP-1) (R&D Systems, Minneapolis, MN, USA). Protein bands were visualized by laser-induced fluorescence using infrared scanner for protein quantification (Odyssey, Li-Cor Biosciences, Lincoln, NE, USA).

### DNA damage kit

A DNA damage kit that determines apurinic/apyrimidinic sites as indicators of DNA lesions by using an Aldehyde Reactive Probe (PromoKine, Heidelberg, Germany) was used following the manufacturer's instructions.

### TP53 next-generation sequencing

All coding exonic and flanking intronic regions of the human TP53 gene were amplified from genomic DNA with Platinum Taq DNA polymerase (Life Technologies) by multiplex PCR, using two primer pools with 12 non-overlapping primer pairs each, yielding ~180 bp amplicons. Each sample was tagged with a unique 8-nucleotide barcode combination using twelve differently barcoded forward and eight differently barcoded reverse primer pools. Barcoded PCR products from up to 96 samples were pooled, purified, and an indexed sequencing library was prepared using the NEBNext ChIP-Seq Library Prep Master Mix Set for Illumina in combination with NEBNext Multiplex Oligos for Illumina (New England Biolabs). The quality of sequencing libraries was verified on a Bioanalyzer DNA High Sensitivity chip (Agilent) and quantified by digital PCR. 2 × 250 bp paired-end sequencing was carried out on an Illumina MiSeq (Illumina) according to the manufacturer's recommendations at a mean coverage of 300 ×.

Read pairs were demultiplexed according to the forward and reverse primers and subsequently aligned using the Burrows-Wheeler Aligner against the Homo sapiens Ensembl reference (rev. 79). Overlapping mate pairs were combined and trimmed to the amplified region. Coverage for each amplicon was calculated via SAMtools (v1.1).^43^ To identify putative mutations, variant calling was performed using SAMtools in combination with VarScan2 (v2.3.9).^44^ Initially, SAMtools was used to create pileups with a base quality filter of 15. Duplicates, orphan reads, unmapped, and secondary reads were excluded. Subsequently, Varscan2 was applied to screen for SNPs and InDels separately, using a low-stringency setting with minimal variant frequency of 0.1, a minimum coverage of 20, and a minimum of 10 supporting reads per variant to account for cellular and clonal heterogeneity. Minimum average quality was set to 20 and a strand filter was applied to minimize miscalls due to poor sequencing quality or amplification bias. The resulting list of putative variants was compared against the IARC TP53 (R17) database to check for known p53 cancer mutations.

### Statistics

Results are expressed as mean±S.D. of at least three experiments. Comparisons between two groups were performed using Student's *t*-test. Three and more groups were compared by ANOVA followed by the Student–Newman–Keuls test. *P* values lower than 0.05 were considered to be significant.

## Abbreviations

ABCB1: ATP Binding Cassette Subfamily B Member 1
ABC transporter: ATP-binding cassette transporter
IAP: inhibitor of apoptosis protein
Chk2: checkpoint kinase 2
*γ*H2AX: *γ*-histone H2A
Mcl-1: Myeloid Cell Leukemia 1
MDM2: Mouse Double Minute 2
MDR1: multidrug resistance gene 1
MYCN: V-Myc Avian Myelocytomatosis Viral Oncogene Neuroblastoma Derived Homolog
PARP: poly(ADP-ribose) polymerase
RCCL collection: resistant cancer cell line collection
SLC35F2: Solute Carrier Family 35 Member F2
XIAP: X-linked inhibitor of apoptosis
