# Supplementary material for: Effects of YM155 on survivin levels and viability in neuroblastoma cells with acquired drug resistance
Source: Cell Death Dis. 2016 Oct 13;7(10):e2410–. doi: 10.1038/cddis.2016.257 (PMC5133961; doi:10.1038/cddis.2016.257)
Supplement: Supplementary Figure 1 [file cddis2016257x1.pdf]

Suppl Figure 1

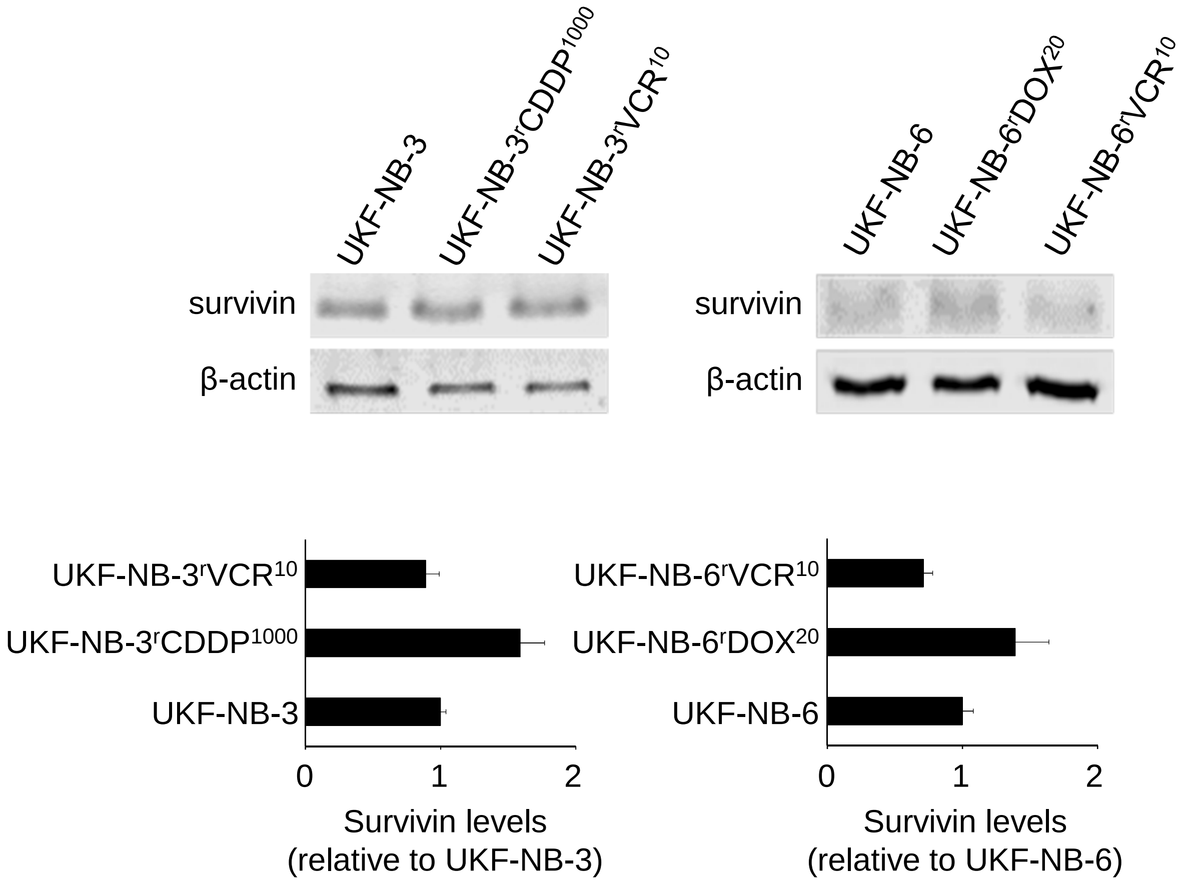

Suppl. Figure 1. Representative Western blots and quantitative fluorescence-based quantification of Western blot data indicating cellular survivin levels in UKF-NB-3 cells, UKF-NB-3 cells with acquired resistance to cisplatin (UKF-NB-3^{r}CDDP^{1000}), UKF-NB-3 cells with acquired resistance to vincristine (UKF-NB-3^{r}VCR^{10}), UKF-NB-6 cells, UKF-NB-6 cells with acquired resistance to doxorubicin (UKF-NB-6^{r}DOX^{20}), or UKF-NB-6 cells with acquired resistance to vincristine (UKF-NB-6^{r}VCR^{10}). β-actin served as loading control.
