# Supplementary material for: Effects of YM155 on survivin levels and viability in neuroblastoma cells with acquired drug resistance
Source: Cell Death Dis. 2016 Oct 13;7(10):e2410–. doi: 10.1038/cddis.2016.257 (PMC5133961; doi:10.1038/cddis.2016.257)
Supplement: Supplementary Figure 2 [file cddis2016257x2.pdf]

Suppl Figure 2

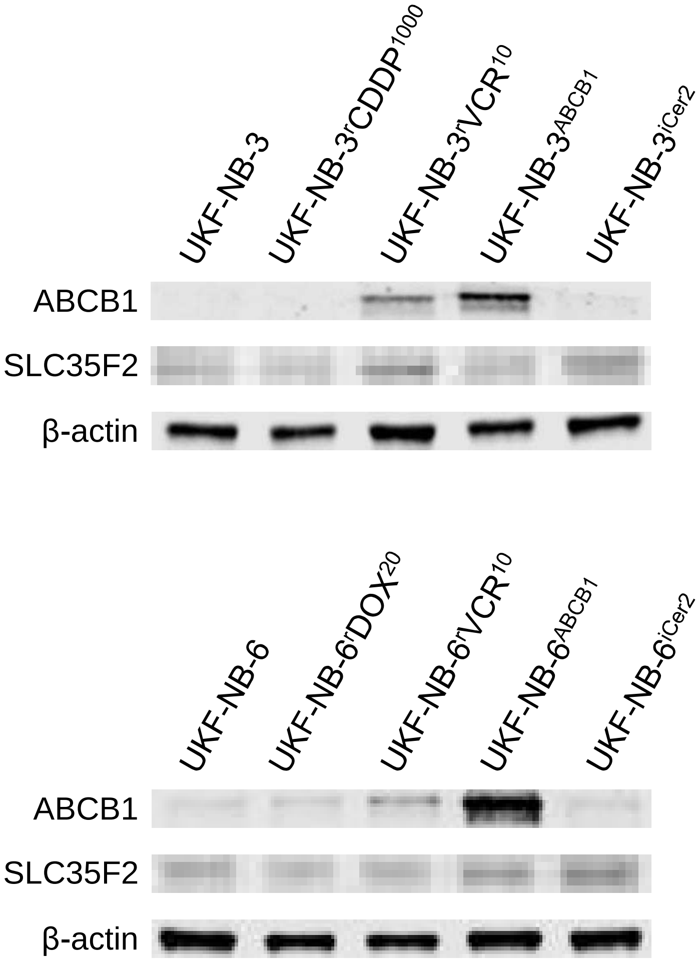

Suppl. Figure 2. Representative Western blots indicating cellular ABCB1 and SLC35F2 levels in UKF-NB-3 cells, UKF-NB-3 cells with acquired resistance to cisplatin (UKF-NB-3^{r}CDDP^{1000}), UKF-NB-3 cells with acquired resistance to vincristine (UKF-NB-3^{r}VCR^{10}), UKF-NB-3 cells transduced with a lentiviral vector encoding for ABCB1 (UKF-NB-3^{ABCB1}), UKF-NB-3 cells transduced with a control vector (UKF-NB-3^{iCer2}), UKF-NB-6 cells, UKF-NB-6 cells with acquired resistance to doxorubicin (UKF-NB-6^{r}DOX^{20}), UKF-NB-6 cells with acquired resistance to vincristine (UKF-NB-6^{r}VCR^{10}), UKF-NB-6 cells transduced with a lentiviral vector encoding for ABCB1 (UKF-NB-6^{ABCB1}), and UKF-NB-6 cells transduced with a control vector (UKF-NB-6^{iCer2}).
