# Supplementary material for: Effects of YM155 on survivin levels and viability in neuroblastoma cells with acquired drug resistance
Source: Cell Death Dis. 2016 Oct 13;7(10):e2410–. doi: 10.1038/cddis.2016.257 (PMC5133961; doi:10.1038/cddis.2016.257)
Supplement: Supplementary Figure 3 [file cddis2016257x3.pdf]

Suppl. Figure 3

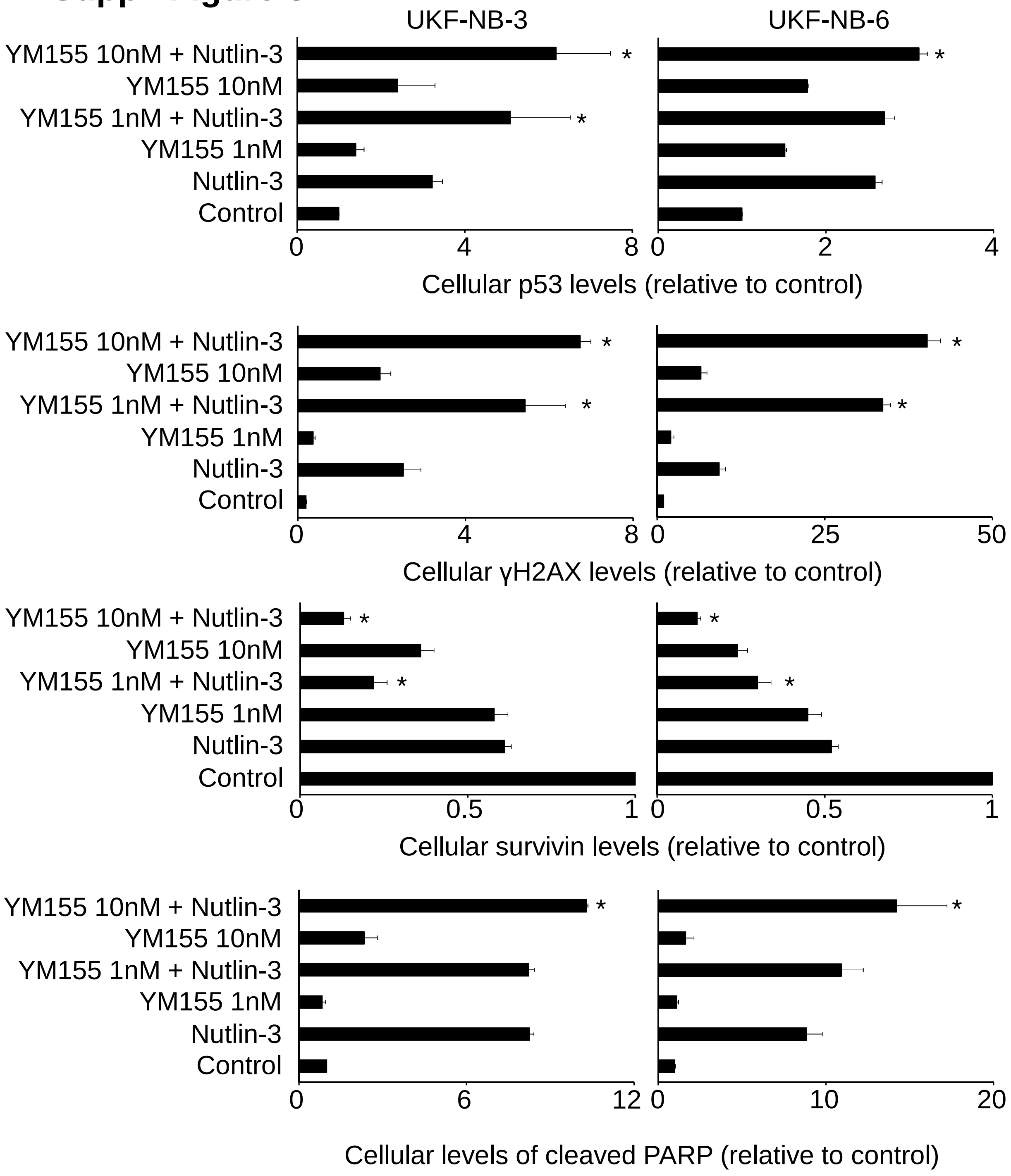

Suppl. Figure 3. Quantitative, fluorescence-based Western blot analysis indicating the levels of p53, γH2AX, survivin, and cleaved PARP in UKF-NB-3 and UKF-NB-6 cells in response to treatment with nutlin-3 (1µM) alone or in combination with YM155. * $P < 0.05$ relative to either single treatment
