# Supplementary material for: Effects of YM155 on survivin levels and viability in neuroblastoma cells with acquired drug resistance
Source: Cell Death Dis. 2016 Oct 13;7(10):e2410–. doi: 10.1038/cddis.2016.257 (PMC5133961; doi:10.1038/cddis.2016.257)
Supplement: Supplementary Figure 4 [file cddis2016257x4.pdf]

# Suppl Figure 4

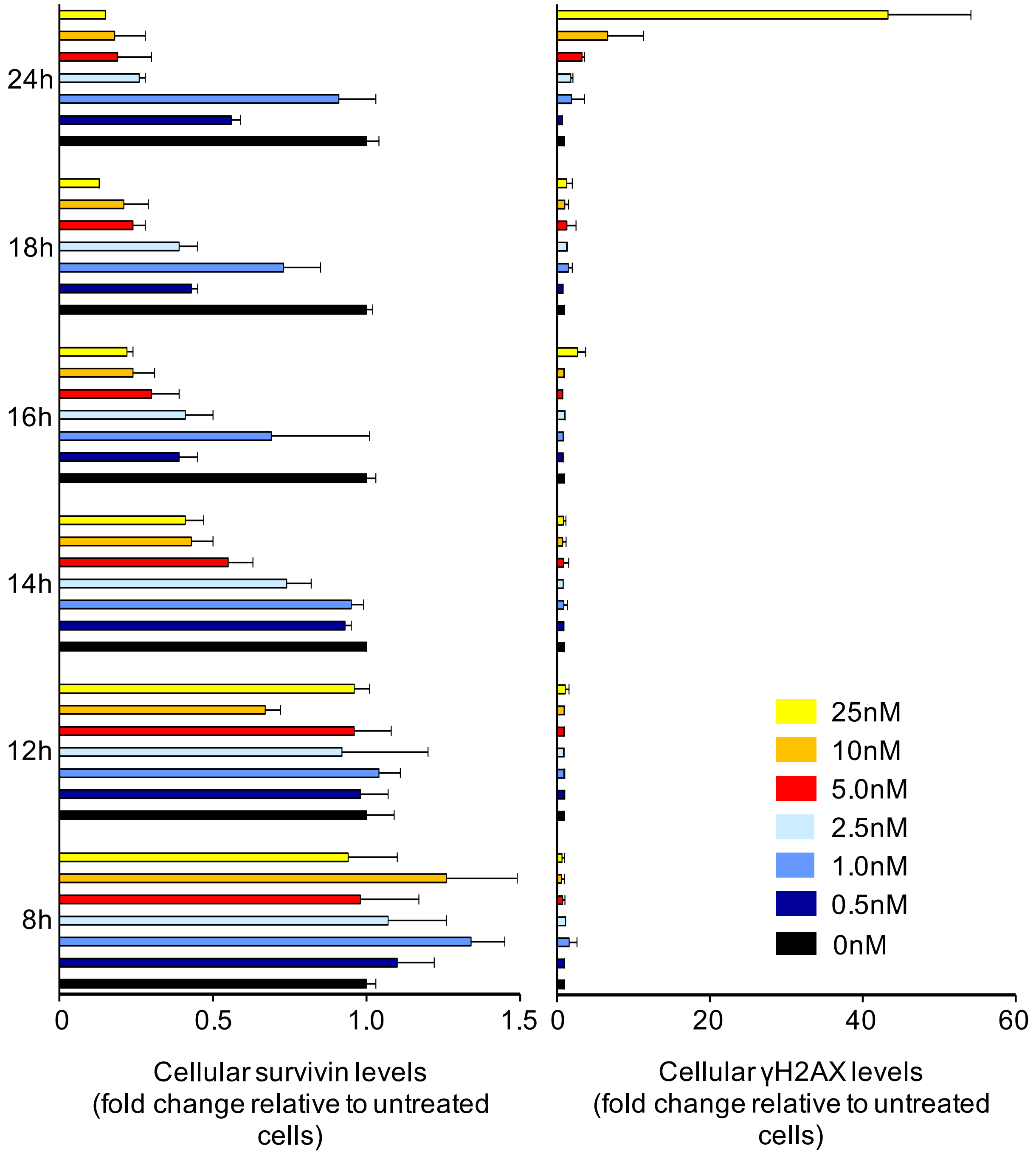


**Suppl. Figure 4A**. Levels of survivin and γH2AX (indicating DNA damage) in UKF-NB-3 cells in response to YM155 treatment over 24h as indicated by quantitative, fluorescence-based Western blot analysis; values are presented as fold change relative to the respective parental cell line.

# Suppl Figure 4

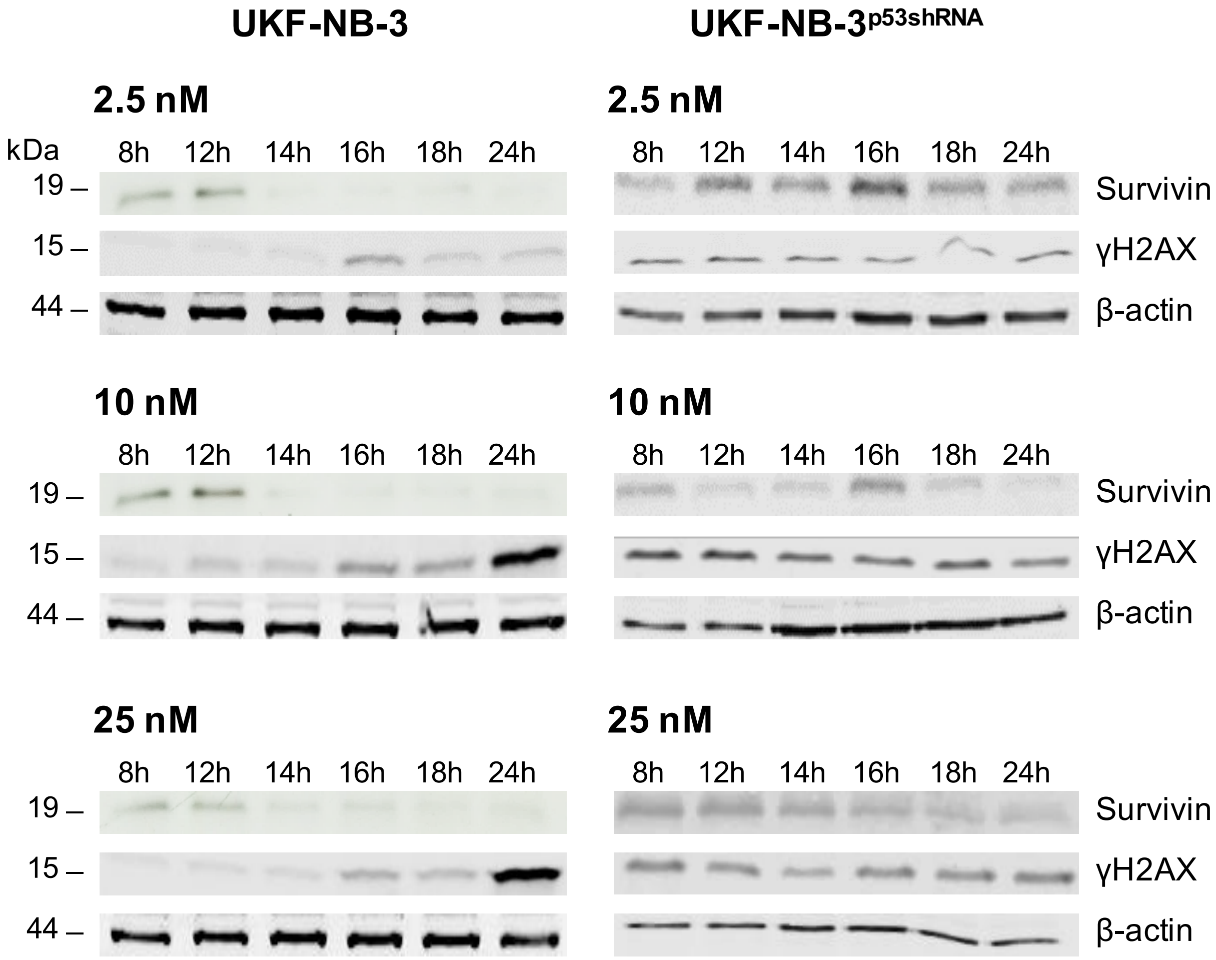


**Suppl. Figure 4B**. Representative Western blots indicating cellular survivin and γH2AX levels in UKF-NB-3 cells or UKF-NB-3 cells transduced with a lentiviral vector encoding for shRNA targeting p53 (UKF-NB-3$^{p53shRNA}$) treated with various YM155 concentrations at different time points.
