# Supplementary material for: Effects of YM155 on survivin levels and viability in neuroblastoma cells with acquired drug resistance
Source: Cell Death Dis. 2016 Oct 13;7(10):e2410–. doi: 10.1038/cddis.2016.257 (PMC5133961; doi:10.1038/cddis.2016.257)
Supplement: Supplementary Figure 5 [file cddis2016257x5.pdf]

Suppl Figure 5

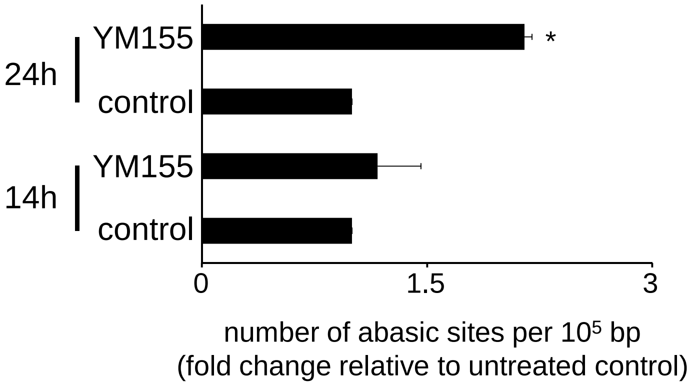

Suppl. Figure 5. DNA damage induced by YM155 (10nM) after 14h or 24h of treatment as indicated by a DNA damage kit that determines apurinic/apyrimidinic sites as indicators of DNA lesions by using an Aldehyde Reactive Probe (PromoKine, Heidelberg, Germany).
