# Supplementary material for: Effects of YM155 on survivin levels and viability in neuroblastoma cells with acquired drug resistance
Source: Cell Death Dis. 2016 Oct 13;7(10):e2410–. doi: 10.1038/cddis.2016.257 (PMC5133961; doi:10.1038/cddis.2016.257)
Supplement: Supplementary Figure 6 [file cddis2016257x6.pdf]

Suppl. Figure 6

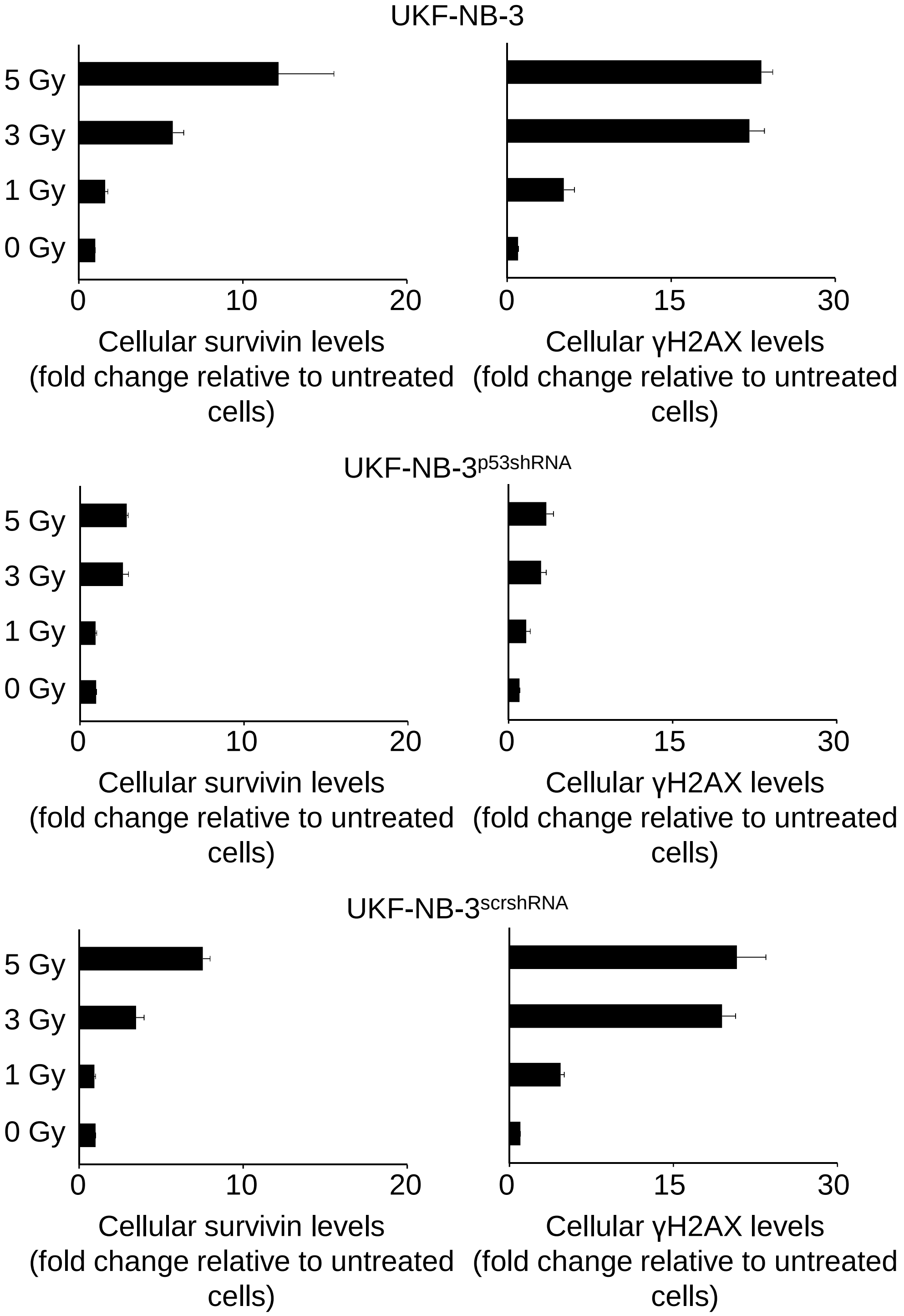

Suppl. Figure 6. Quantitative, fluorescence-based Western blot analysis of the survivin and γH2AX levels in UKF-NB-3 cells, UKF-NB-3 cells transduced with a lentiviral vector encoding p53 shRNA (UKF-NB-3^{p53shRNA}), or UKF-NB-3 cells transduced with a lentiviral vector encoding non-targeting (‘scrambled’) shRNA (UKF-NB-3^{scrshRNA}) 24h post irradiation.
