# Supplementary material for: Effects of YM155 on survivin levels and viability in neuroblastoma cells with acquired drug resistance
Source: Cell Death Dis. 2016 Oct 13;7(10):e2410–. doi: 10.1038/cddis.2016.257 (PMC5133961; doi:10.1038/cddis.2016.257)
Supplement: Supplementary Figure 7 [file cddis2016257x7.pdf]

Suppl. Figure 7

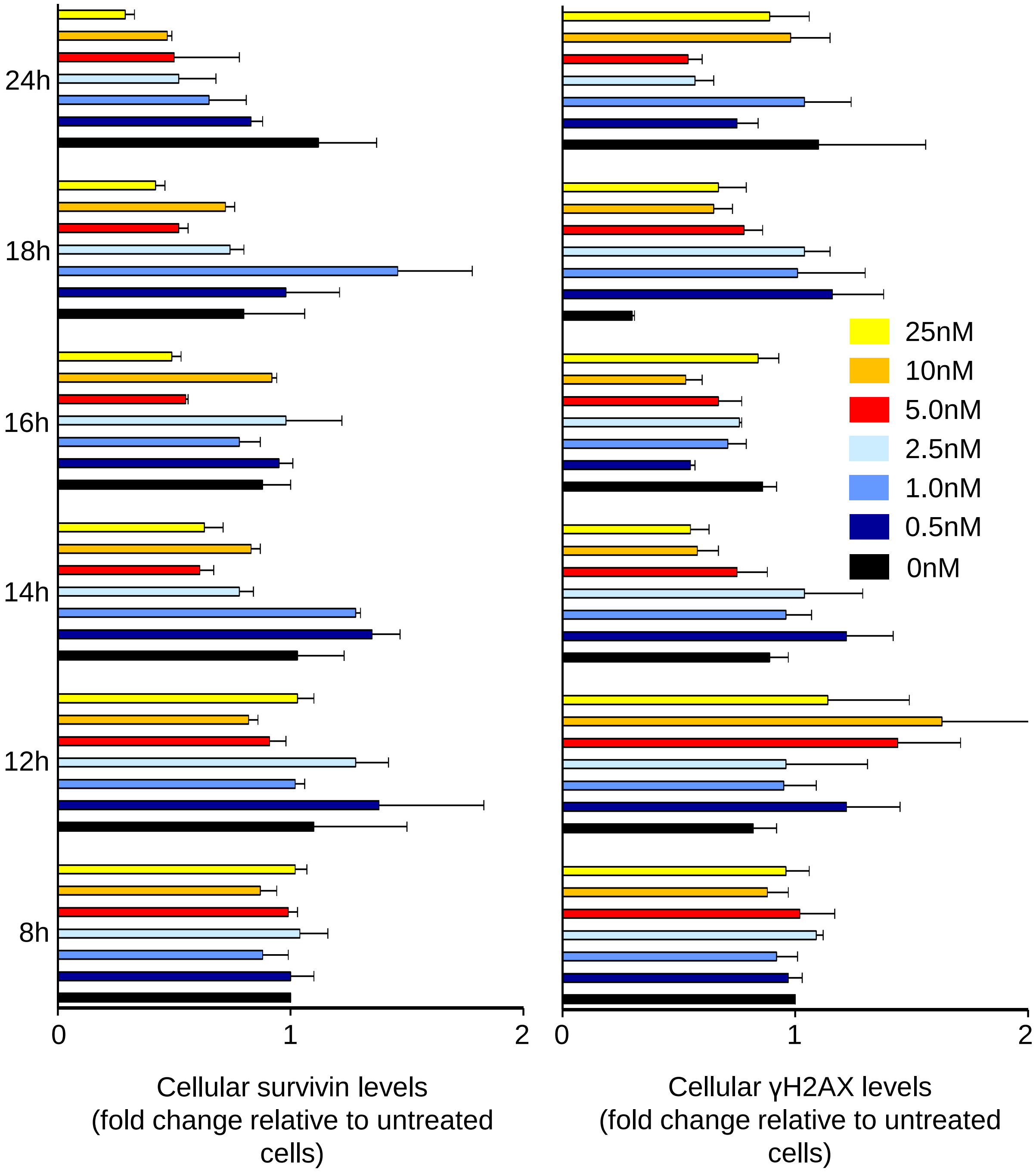

Suppl. Figure 7. Kinetics of survivin depletion and DNA damage induction by YM155 in neuroblastoma cells. Cellular levels of survivin and γH2AX (indicating DNA damage) in UKF-NB-3^{p53shRNA} cells in response to YM155 treatment over 24h as indicated by quantitative, fluorescence-based Western blot analysis; values are presented as fold change relative to the respective parental cell line. Representative Western blots are provided in Suppl. Figure 4.
