# Supplementary material for: Effects of YM155 on survivin levels and viability in neuroblastoma cells with acquired drug resistance
Source: Cell Death Dis. 2016 Oct 13;7(10):e2410–. doi: 10.1038/cddis.2016.257 (PMC5133961; doi:10.1038/cddis.2016.257)
Supplement: Supplementary Figure 8 [file cddis2016257x8.pdf]

Suppl Figure 8

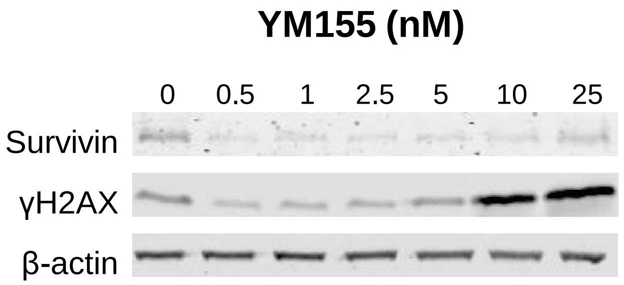

Suppl. Figure 8. Representative Western blots indicating cellular survivin and γH2AX levels in UKF-NB-3 cells transduced with a lentiviral vector encoding for shRNA targeting p53 (UKF-NB-3^{p53shRNA}) treated with various YM155 concentrations for 48h.
