# Supplementary material for: Effects of YM155 on survivin levels and viability in neuroblastoma cells with acquired drug resistance
Source: Cell Death Dis. 2016 Oct 13;7(10):e2410–. doi: 10.1038/cddis.2016.257 (PMC5133961; doi:10.1038/cddis.2016.257)
Supplement: Supplementary Figure 9 [file cddis2016257x9.pdf]

Suppl Figure 9

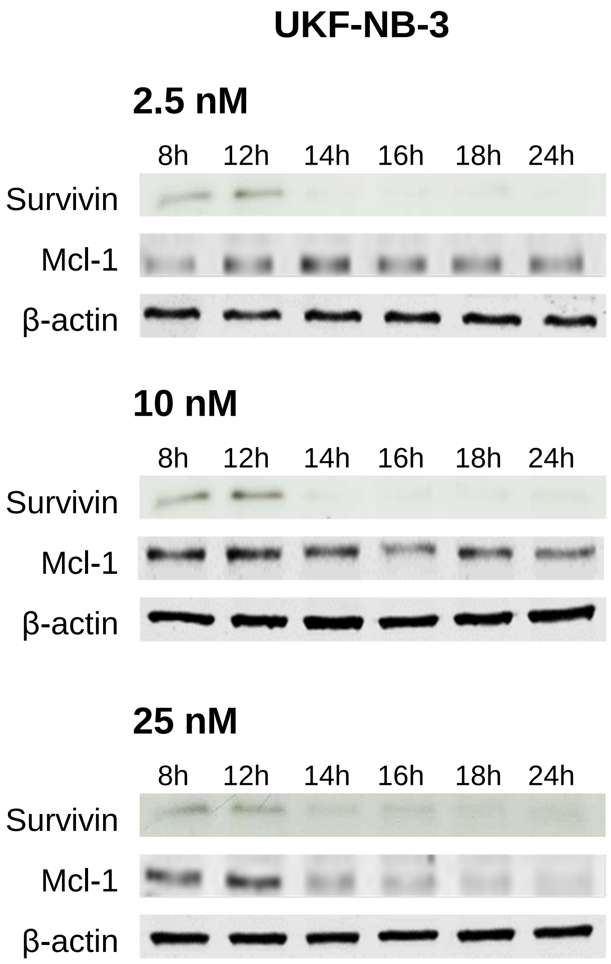

Suppl. Figure 9. Representative Western blots indicating survivin and Mcl-1 levels in UKF-NB-3 cells treated with various YM155 concentrations at different time points.
