# Supplementary material for: Effects of YM155 on survivin levels and viability in neuroblastoma cells with acquired drug resistance
Source: Cell Death Dis. 2016 Oct 13;7(10):e2410–. doi: 10.1038/cddis.2016.257 (PMC5133961; doi:10.1038/cddis.2016.257)
Supplement: Supplementary Figure 10 [file cddis2016257x10.pdf]

Suppl Figure 10

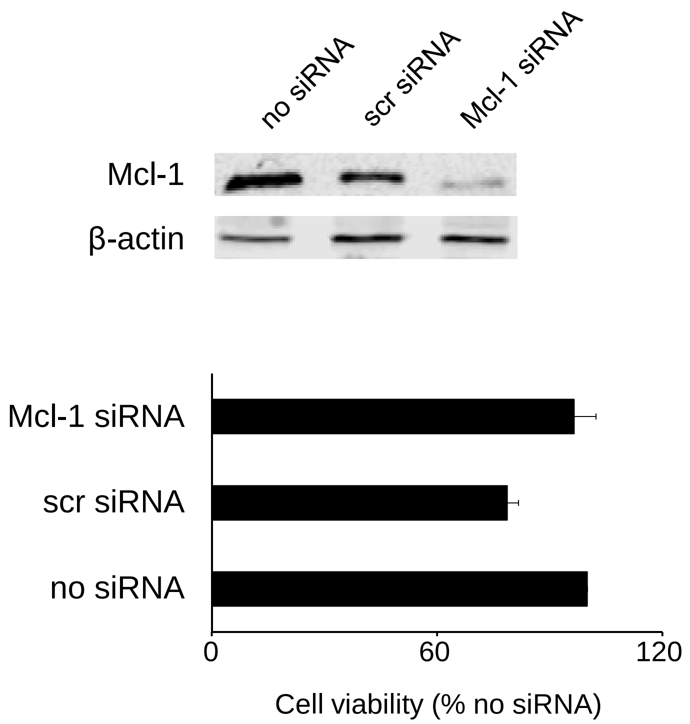

Suppl. Figure 10. Effects of RNAi-mediated Mcl-1 depletion on UKF-NB-3 cells. UKF-NB-3 cells were treated without siRNA, with non-targeting ‘scrambled’ (scr) siRNA, or siRNA directed against Mcl-1. Cellular Mcl-1 levels were detected 24h post transfection by Western blot. Cell viability was determined 72h post transfection by MTT assay.
