# Supplementary material for: Effects of YM155 on survivin levels and viability in neuroblastoma cells with acquired drug resistance
Source: Cell Death Dis. 2016 Oct 13;7(10):e2410–. doi: 10.1038/cddis.2016.257 (PMC5133961; doi:10.1038/cddis.2016.257)
Supplement: Supplementary Figure 11 [file cddis2016257x11.pdf]

Suppl Figure 11

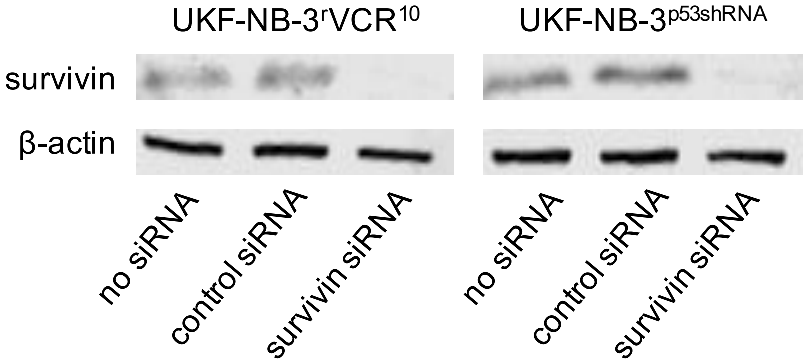

Suppl. Figure 11. Western blots indicating levels survivin in UKF-NB-3^{r}VCR^{10} cells or UKF-NB-3 cells transduced with a lentiviral vector encoding shRNA directed against p53 (UKF-NB-3^{p53shRNA}) 24h post transfection with siRNA targeting survivin (BIRC5). β-actin served as loading control.
