# Supplementary material for: Effects of YM155 on survivin levels and viability in neuroblastoma cells with acquired drug resistance
Source: Cell Death Dis. 2016 Oct 13;7(10):e2410–. doi: 10.1038/cddis.2016.257 (PMC5133961; doi:10.1038/cddis.2016.257)
Supplement: Supplementary Table 1 [file cddis2016257x12.pdf]

Suppl. Table 1. YM155 concentrations that reduce neuroblastoma cell viability by 50% (IC50) after 120h of incubation in the absence or presence of the ABCB1 inhibitor verapamil as indicated by MTT assay.

		+ verapamil (5µM)	
Cell line	YM155 IC50 (nM)	verapamil alone (% untreated control)	YM155 IC50 (nM)
UKF-NB-3	0.49 ± 0.10	102 ± 13	0.61 ± 0.13
UKF-NB-3^{r}CDDP^{1000}	5.32 ± 1.21	91 ± 9	13.85 ± 1.24
UKF-NB-3^{r}VCR^{10}	26.59 ± 6.37	104 ± 8	1.95 ± 0.28
UKF-NB-6	0.65 ± 0.09	95 ± 17	0.58 ± 0.07
UKF-NB-6^{r}DOX^{20}	11.80 ± 1.56	97 ± 10	1.35 ± 0.35
UKF-NB-6^{r}VCR^{10}	49.30 ± 2.24	109 ± 14	3.60 ± 0.12
