# Supplementary material for: Effects of YM155 on survivin levels and viability in neuroblastoma cells with acquired drug resistance
Source: Cell Death Dis. 2016 Oct 13;7(10):e2410–. doi: 10.1038/cddis.2016.257 (PMC5133961; doi:10.1038/cddis.2016.257)
Supplement: Supplementary Table 2 [file cddis2016257x13.pdf]

**Suppl. Table 2**. YM155 concentrations that reduce the viability of UKF-NB-3 cells, UKF-NB-3 cells transduced with a lentiviral control vector (UKF-NB-3$^{sh\ control}$), and UKF-NB-3 cells transduced with lentiviral vectors encoding for shRNAs targeting different sequences of SLC35F2 (UKF-NB-3$^{sh1\ SLC35F2}$, UKF-NB-3$^{sh2\ SLC35F2}$, UKF-NB-3$^{sh3\ SLC35F2}$) by 50% (IC50) after 120h of incubation as indicated by MTT assay.

| Cell line | YM155 IC50 (nM) |
| --- | --- |
| UKF-NB-3 | 0.48 ± 0.02 |
| UKF-NB-3$^{sh\ control}$ | 0.58 ± 0.03 |
| UKF-NB-3$^{sh1\ SLC35F2}$ | 4.10 ± 0.01 |
| UKF-NB-3$^{sh2\ SLC35F2}$ | 3.63 ± 1.22 |
| UKF-NB-3$^{sh3\ SLC35F2}$ | 4.85 ± 0.07 |
