# Supplementary material for: Effects of YM155 on survivin levels and viability in neuroblastoma cells with acquired drug resistance
Source: Cell Death Dis. 2016 Oct 13;7(10):e2410–. doi: 10.1038/cddis.2016.257 (PMC5133961; doi:10.1038/cddis.2016.257)
Supplement: Supplementary Table 3 [file cddis2016257x14.pdf]

Suppl. Table 3. YM155 concentrations that reduce the viability of UKF-NB-3 cells or ABCB1-transduced UKF-NB-3 cells (UKF-NB-3^{ABCB1}) after transfection with siRNA targeting SLC35F2 or non-targeting control siRNA by 50% (IC50) after 120h of incubation as indicated by MTT assay.

Cell line	YM155 IC50 (nM)
UKF-NB-3	0.56 ± 0.03
UKF-NB-3$^{non\text{-}targeting\ siRNA}$	0.57 ± 0.05
UKF-NB-3$^{SLC35F2\ siRNA}$	2.03 ± 0.19
UKF-NB-3^{ABCB1}	2055 ± 95
UKF-NB-3$^{ABCB1\ non\text{-}targeting\ siRNA}$	2012 ± 173
UKF-NB-3$^{ABCB1\ SLC35F2\ siRNA}$	3465 ± 211
