# Supplementary material for: Effects of YM155 on survivin levels and viability in neuroblastoma cells with acquired drug resistance
Source: Cell Death Dis. 2016 Oct 13;7(10):e2410–. doi: 10.1038/cddis.2016.257 (PMC5133961; doi:10.1038/cddis.2016.257)
Supplement: Supplementary Table 4 [file cddis2016257x15.pdf]

Suppl. Table 4. YM155 concentrations that reduce the viability of UKF-NB-3 cells or UKF-NB-6 cells, UKF-NB-3 or UKF-NB-6 cell transduced with a control vector encoding for non-targeting ('scambled') shRNA (UKF-NB-3^{scrshRNA}, UKF-NB-6^{scrshRNA}), or UKF-NB-3 or UKF-NB-6 cells transduced with lentiviral vector encoding for shRNA targeting p53 (UKF-NB-3^{p53shRNA}, UKF-NB-6^{p53shRNA}) as well as UKF-NB-3 cells adapted to nutlin-3 (UKF-NB-3^{r}Nutlin$^{10\mu M}$) harboring a G245C p53 loss-of-function mutation by 50% (IC50) after 120h of incubation as indicated by MTT assay.

Cell line	YM155 IC50 (nM)
UKF-NB-3	0.49 ± 0.10
UKF-NB-3^{scrshRNA}	0.50 ± 0.14
UKF-NB-3^{p53shRNA}	4.30 ± 0.97
UKF-NB-3^{r}Nutlin$^{10\mu M}$	1.18 ± 0.07
UKF-NB-6	0.74 ± 0.04
UKF-NB-6^{scrshRNA}	0.52 ± 0.08
UKF-NB-6^{p53shRNA}	4.23 ± 0.11
