# Supplementary material for: Effects of YM155 on survivin levels and viability in neuroblastoma cells with acquired drug resistance
Source: Cell Death Dis. 2016 Oct 13;7(10):e2410–. doi: 10.1038/cddis.2016.257 (PMC5133961; doi:10.1038/cddis.2016.257)
Supplement: Supplementary Table 5 [file cddis2016257x16.pdf]

Suppl. Table 5. Viability of neuroblastoma cell lines 48h after transduction with non-targeting siRNA or siRNA targeting survivin relative to untreated control.

	survivin (BIRC5) siRNA	non-targeting siRNA
Cell line	Cell viability (% untreated control)	Cell viability (% untreated control)
UKF-NB-3	0	100.87 ± 3.01
UKF-NB-3^{r}VCR^{10}	23.21 ± 2.63	103.30 ± 3.64
UKF-NB-3^{p53shRNA}	10.93 ± 0.87	97.35 ± 5.70
UKF-NB-3^{r}YM155^{20nM}	25.20 ± 9.86	103.03 ± 19.01
