# Supplementary material for: Effects of YM155 on survivin levels and viability in neuroblastoma cells with acquired drug resistance
Source: Cell Death Dis. 2016 Oct 13;7(10):e2410–. doi: 10.1038/cddis.2016.257 (PMC5133961; doi:10.1038/cddis.2016.257)
Supplement: Supplementary Table 6 [file cddis2016257x17.pdf]

Suppl. Table 6. YM155 concentrations that reduce neuroblastoma cell viability by 50% (IC50) after 120h of incubation in the absence or presence of the ABCB1 inhibitor verapamil as indicated by MTT assay.

		+ verapamil (5µM)	
Cell line	YM155 IC50 (nM)	verapamil alone (% untreated control)	YM155 IC50 (nM)
UKF-NB-3	0.61 ± 0.08	95 ± 9	0.65 ± 0.13
UKF-NB-3^{r}YM155^{20nM}	302.55 ± 7.57	96 ± 5	90.54 ± 3.14
